# 2011 German *Escherichia coli *O104:H4 outbreak: whole-genome phylogeny without alignment

**DOI:** 10.1186/1756-0500-4-533

**Published:** 2011-12-13

**Authors:** Man Kit Cheung, Lei Li, Wenyan Nong, Hoi Shan Kwan

**Affiliations:** 1 School of Life Sciences, The Chinese University of Hong Kong, Shatin, New Territories, Hong Kong SAR, China

## Abstract

**Background:**

A large-scale *Escherichia coli *O104:H4 outbreak occurred in Germany from May to July 2011, causing numerous cases of hemolytic-uremic syndrome (HUS) and deaths. Genomes of ten outbreak isolates and a historical O104:H4 strain isolated in 2001 were sequenced using different new generation sequencing platforms. Phylogenetic analyses were performed using various approaches which either are not genome-wide or may be subject to errors due to poor sequence alignment. Also, detailed pathogenicity analyses on the 2001 strain were not available.

**Findings:**

We reconstructed the phylogeny of *E. coli *using the genome-wide and alignment-free feature frequency profile method and revealed the 2001 strain to be the closest relative to the 2011 outbreak strain among all available *E. coli *strains at present and confirmed findings from previous alignment-based phylogenetic studies that the HUS-causing O104:H4 strains are more closely related to typical enteroaggregative *E. coli *(EAEC) than to enterohemorrhagic *E. coli*. Detailed re-examination of pathogenicity-related virulence factors and secreted proteins showed that the 2001 strain possesses virulence factors shared between typical EAEC and the 2011 outbreak strain.

**Conclusions:**

Our study represents the first attempt to elucidate the whole-genome phylogeny of the 2011 German outbreak using an alignment-free method, and suggested a direct line of ancestry leading from a putative EAEC-like ancestor through the 2001 strain to the 2011 outbreak strain.

## Background

In early May 2011, a large outbreak of diarrhea with associated hemolytic-uremic syndrome (HUS) began in Germany. Until its official end in late July, 782 cases of HUS (29 deaths) and 3128 non-HUS cases (17 deaths) were reported, making it the largest outbreak of HUS worldwide [1]. Diarrhea associated with HUS is usually caused by enterohemorrhagic *E. coli *(EHEC) [2]. However, the outbreak strain was serotyped to be O104:H4, which historically caused very few HUS cases [3]. Early PCR assay and cell-adherence assay revealed genotypic and phenotypic characteristics of enteroaggregative *E. coli *(EAEC) [4]. In order to characterize the unusual strain, genomes of ten outbreak isolates were sequenced using next-generation and third-generation sequencing technologies [5-8]. The genome sequence of a historical O104:H4 strain, 01-09591, isolated in 2001 [9] was also obtained [8].

Phylogenetic analyses were performed to understand the evolution of the outbreak strain using various approaches [5,6,8,10]. However, the multilocus sequence analysis (MLSA)-based approach used information from only seven housekeeping genes [5], the study of Mellmann et al. [8] employed only core protein-coding genes and the single nucleotide polymorphism (SNP)-based approach might suffer from wrong SNP calling [11]. In addition, accuracy of most of these methods relies on the quality of sequence alignment. Based on compositions of *l*-mer features of whole genomes, the feature frequency profile (FFP) method is alignment-free and truly genome-wide [12]. It has been successfully applied on resolving relationships among *E. coli *strains [13] and mammals [14]. In this study, we reconstructed the whole-genome phylogeny of *E. coli *using the FFP approach. Pathogenicity-related virulence factors and secreted proteins of the historical O104:H4 strain were also re-examined in detail, aiming to better understand its relationship with, and probably the evolution of, the 2011 outbreak strain.

### Phylogenetic analysis

**Figure 1:**
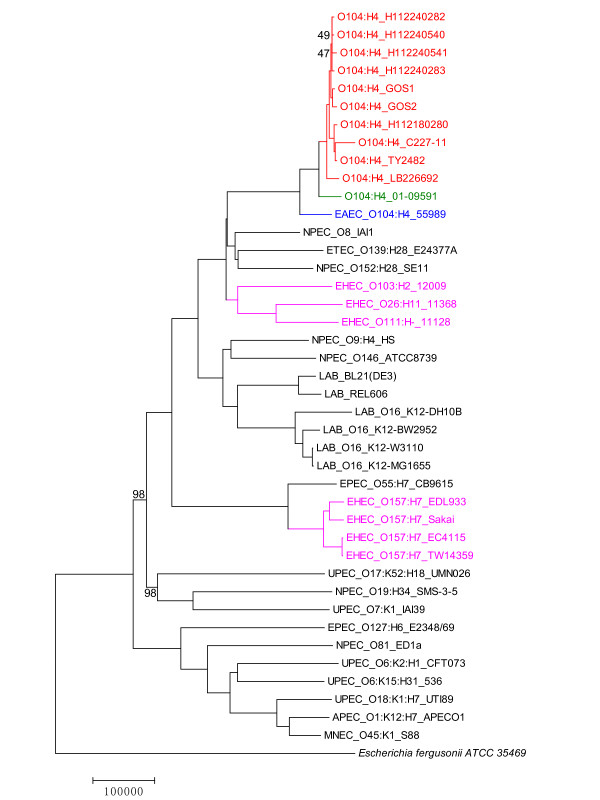
**Whole-genome phylogenetic tree of *E. coli***. The FFP tree is based on 1,125,665 low-frequency core features shared among all 42 isolates. *E. coli *isolates were named in the format of pathotype followed by serotype, if known, and then isolate name. The 2011 German outbreak isolates were highlighted in red, the 2001 strain in green, the EAEC strain in blue and typical EHEC strains in pink. Branch length represents the number of character feature changes. Numerical values at nodes are 10% jackknife confidence values, no value represents 100% agreement among pseudoreplicates. *E. fergusonii *ATCC 35469 was used as the outgroup. Abbreviations of pathotypes are: enterohemorrhagic *E. coli *(EHEC), enteroaggregative *E. coli *(EAEC), non-pathogenic *E. coli *(NPEC), enterotoxigenic *E. coli *(ETEC), uropathogenic *E. coli *(UPEC), enteropathogenic *E. coli *(EPEC), avian pathogenic *E. coli *(APEC) and meningitis-associated *E. coli *(MNEC).

Based on genome sequences of ten *E. coli *isolates from the 2011 German O104:H4 outbreak, the historical O104:H4 strain 01-09591 and 30 additional *E. coli *strains, we tried to infer the lineal phylogeny of *E. coli *using the alignment-free FFP method (Figure 1). It is well known that lateral transfer of mobile elements is common in *E. coli *[15], and it can make lineal phylogenetic inference difficult. In this study, in order to reduce the effect of lateral gene transfer on tree topology, we 1) extracted only core features that were present in all *E. coli *strains, 2) removed features likely to be associated with mobile or repetitive DNA by filtering out features with high frequencies, and 3) calculated genetic distances among *E. coli *strains using an unordered character state model [16], following the approach as described in Sims & Kim [13].

A close relationship among all the ten German outbreak isolates was revealed in our phylogenetic trees (Figure 1, Additional file 1). The overall high similarity among the outbreak isolates agrees with previous reports using whole-chromosome optical maps [8] and SNPs [5,6,10], suggesting a clonal and probably single-sourced nature of the outbreak. Our FFP tree also revealed a cluster formed between 01-09591 and the 2011 outbreak isolates, which is distantly related to other typical EHEC strains, and with EAEC 55989 placed in a basal position to the two lineages (Figure 1). Before the genome sequence of the 2001 strain is made available, EAEC 55989, which is another O104:H4 strain isolated from a patient in Central Africa in late 1990s [17], was shown to be the closest relative of the 2011 outbreak strain among all available *E. coli *genomes in early phylogenetic analyses based on SNPs [10] and MLSA [5]. In the study in which the 2001 strain was sequenced, a higher relatedness of the 2011 outbreak isolates with 01-09591 than to EAEC 55989 was suggested by whole-chromosome optical map similarity clustering analysis based on restriction patterns [8]. Our FFP tree provides another piece of evidence to this, suggesting that 01-09591 isolated in 2001 is the closest relative to the 2011 outbreak strain, among all available *E. coli *genomes at present. Based on an alignment-free principle, our FFP tree also confirms findings from previous alignment-based phylogenetic studies that the 2011 outbreak strain, and the HUS-causing O104:H4 group, is more closely related to typical EAEC than to EHEC strains [5,6,8,10].

### Pathogenicity analyses

Pathogenicity-related virulence factors and secreted proteins of 01-09591 isolated in 2001 were compared with those of three isolates from the 2011 outbreak, TY2482, H112180280 and C227-11, and representatives from typical EAEC and EHEC. The three outbreak isolates were selected based on their relative complete assembly of genomes compared to the other isolates (Additional file 2). Our analyses showed that the 2001 strain shared many common features with the 2011 outbreak strain, in terms of virulence factors and secreted proteins (Additional files 3 and 4). For instance, both of them possess the Shiga toxin-encoding *stx2 *genes, which are typically harbored by EHEC; however, both of them lack gene clusters coding for EHEC-type type III secretion system (TTSS). The absence of *eae *and *ler *in both strains provides additional piece of evidence for the absence of the locus of enterocyte effacement (LEE) pathogenicity island, which is responsible for attachment to the intestine in typical EHEC [2]. Indeed, except *stx2*, other virulence factors involved in EHEC-type iron uptake and encoding EHEC-type toxin are missing in both strains. Instead, both strains harbor the full set of dispersin-related genes of typical EAEC and the whole set of EAEC-specific protein secretion genes.

However, our analyses also showed that the 2001 strain harbors virulence factors that resemble EAEC 55989 more than to the 2011 outbreak strain (Additional file 3). For instance, both the 2001 strain and EAEC 55989 harbor *agg3A-D *which encode type III aggregative adherence fimbriae (AAF/III) instead of *aggA-D *encoding AAF/I as in the 2011 outbreak strain. In addition, besides *pic *and *set1A-B*, the 2001 strain also possesses *astA *that codes for an extra EAEC-type toxin, just like EAEC 55989. These showed that the 2001 strain is already an unusual pathotype with genotypic characteristics of both EAEC and EHEC, which possesses virulence factors shared between EAEC 55989 and the 2011 outbreak strain.

Results of our phylogenetic analysis and pathogenicity analyses together suggested the 2001 strain to be some kind of intermediate form between the 2011 outbreak strain and its putative EAEC 55989-like ancestor. Recently, additional historical isolates of HUS-causing *E. coli *O104:H4 were characterized [18-20]. However, genome sequences of these isolates are not available. It is expected that sequencing and analyzing genomes of these and other related or historical isolates would provide further insight about the true evolutionary pathway of the 2011 outbreak strain, as well as that of the unusual HUS-causing *E. coli *O104:H4 group formed by it and the 2001 strain.

## Conclusions

By reconstructing the whole-genome phylogeny of *E. coli *using the alignment-free FFP method, we revealed 01-09591 isolated in 2001 to be the closest relative to the 2011 German O104:H4 outbreak strain among all available *E. coli *strains at present and confirmed findings from previous alignment-based phylogenetic studies that the HUS-causing O104:H4 strains are more closely related to typical EAEC than to EHEC strains. Detailed re-examination of pathogenicity-related virulence factors and secreted proteins showed that the 2001 strain possesses virulence factors shared between EAEC 55989 and the 2011 outbreak strain. A direct line of ancestry leading from an EAEC 55989-like ancestor through the 2001 strain to the 2011 outbreak strain is suggested. However, it is expected that sequencing and analyzing the genomes of other related or historical isolates might help deciphering the true evolutionary history of the 2001 German outbreak.

## Materials and methods

### Sequence data

Genome sequences of ten *E. coli *isolates from the 2011 German O104:H4 outbreak and a historical O104:H4 strain (01-09591) isolated in 2001 were downloaded from GenBank and some private domains (Additional file 2). Complete genome sequences of 30 additional *E. coli *strains [K12-W3110 (AC_000091), K12-MG1655 (NC_000913), EDL933 (NC_002655), Sakai (NC_002695), CFT073 (NC_004431), UTI89 (NC_007946), 536 (NC_008253), APEC01 (NC_008563), HS (NC_009800), E24377A (NC_009801), ATCC8739 (NC_010468), K12-DH10B (NC_010473), SMS-3-5 (NC_010498), EC4115 (NC_011353), SE11 (NC_011415), E2348/69 (NC_011601), IAI1 (NC_011741), S88 (NC_011742), ED1a (NC_011745), 55989 (NC_011748), IAI39 (NC_011750), UMN026 (NC_011751), K12-BW2952 (NC_012759), BL21(DE3) (NC_012947), REL606 (NC_012967), TW14359 (NC_013008), 12009 (NC_013353), 11368 (NC_013361), 11128 (NC_013364), CB9615 (NC_013941)] and that of *E. fergusonii *[ATCC 35469 (NC_011740] were also obtained from GenBank.

### Phylogenetic analysis

Whole-genome phylogeny of *E. coli *was inferred using the alignment-free FFP method as described in Sims & Kim [13] using Perl scripts. In brief, only main chromosome data were used; for those unmapped genomes, BLASTn searches were performed and those contigs returned with plasmids as top hits were removed (E-value ≤1e-5). The genome sequences were then converted into an RY (purine/pyrimidine)-coded form to reduce base composition bias [21] and computational memory requirement. A further reduction of computer resource burden was achieved by considering the forward and reverse complement features equivalent. Feature frequency profiles were established by running a sliding window of length *l *through the whole genomes from position 1 to n - *l *+ 1, with an offset of one nucleotide between windows. The sliding window is not allowed to span over gaps between contigs. The optimal value of *l *was chosen to be 24 using the criterion of topological convergence [14]: as *l *increased, tree topologies converged to a single topology. In this case, tree topologies at *l *= 22 and *l *= 23 are the same as at *l *= 24, but become more divergent beyond the range. In order to avoid the effects of mobile elements on tree topology, only core features that present in all *E. coli *isolates were extracted and high-frequency features occurring more than 3 times, as derived using an extreme-value cumulative-distribution function, in any of the isolates were removed. Also, numerical count was treated as a feature state and simple cumulative distances were calculated among all feature states in an unordered manner. Different states add 1 to the distance, whereas identical states add nothing. Neighbor-joining trees were plotted with the resulting distance matrix using MEGA5 [22]. Statistical confidence on the inferred tree topology was assessed by a 10% jackknife procedure with 100 pseudoreplicates.

### Pathogenicity analyses

EHEC- and EAEC-specific virulence factors were extracted from the Virulence Factor Database [23] and then BLASTed against genome sequences of isolates TY2482, H112180280 and C227-11 from the 2011 outbreak, the 2001 isolate 01-09591, EAEC 55989 and EHEC Sakai. Presence/absence of the genes was determined by the alignment length of top hits in BLAST with an E-value threshold of 1e-5: an alignment length ≥ 50% represents presence of gene, an alignment length < 50% and > 0% represents potential truncation or internal rearrangement of gene, absence of gene was determined when no hit returned. Sequences of regions annotated as secreted proteins in genomes of EAEC 55989 and EHEC Sakai were also extracted and BLASTed against chromosomal genome sequences of the six isolates for presence-absence data using the same set of criteria as the virulence factor analysis.

## Competing interests

The authors declare that they have no competing interests.

## Authors' contributions

MKC carried out the phylogenetic analysis and wrote the manuscript. LL performed the pathogenicity analyses. WN participated in the phylogenetic analysis. HSK conceived and supervised the study. All authors read and approved the final manuscript.

## Supplementary Material

Additional file 1 **Figure S1**. Whole-genome phylogenetic tree of HUS-causing O104:H4. Description: The FFP tree is based on 3,163,595 low-frequency core features shared among all 12 isolates. EAEC 55989 was used as the outgroup. Other definitions as in Figure 1. Click here for file

Additional file 2 **Table S1**. Detailed information of O104:H4 isolates included in this study. Description: Includes information about strain isolation and genome sequencing. Click here for file

Additional file 3 **Table S2**. Detailed results of the virulence factor analysis. Description: Presence-absence data of virulence factor-related genes in the six isolates under investigation. Click here for file

Additional file 4 **Table S3**. Detailed results of the secreted protein analysis. Description: Presence-absence data of protein secretion-related genes in the six isolates under investigation. Click here for file
