# Supplementary figures and images for: 2011 German Escherichia coli O104:H4 outbreak: whole-genome phylogeny without alignment
Source: BMC Res Notes. 2011 Dec 13;4:533. doi: 10.1186/1756-0500-4-533 (PMC3280199; doi:10.1186/1756-0500-4-533)

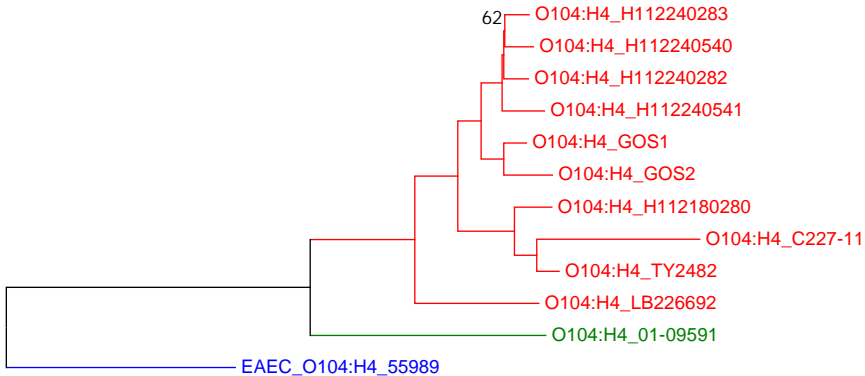

Supplement: Additional file 1 — Figure S1. Whole-genome phylogenetic tree of HUS-causing O104:H4. Description: The FFP tree is based on 3,163,595 low-frequency core features shared among all 12 isolates. EAEC 55989 was used as the outgroup. Other definitions as in Figure 1. [file 1756-0500-4-533-S1.PDF]
